# Antimicrobial treatment of egg-infested olives is associated with perturbation and carry-over changes on bacterial communities in the olive fly *Bactrocera oleae* (Diptera, Tephritidae)

**DOI:** 10.1093/jisesa/ieag108

**Published:** 2026-10-03

**Authors:** Laura Maretto, Ivana Carofano, Isabel Martinez-Sañudo, Marco Falasco, Hannes Schuler, Markus Riegler, Jennifer Morrow, Luca Mazzon

**Affiliations:** Department of Agronomy, Food, Natural Resources, Animals and Environment—DAFNAE, University of Padova, Legnaro, Italy; Department of Agronomy, Food, Natural Resources, Animals and Environment—DAFNAE, University of Padova, Legnaro, Italy; Department of Agronomy, Food, Natural Resources, Animals and Environment—DAFNAE, University of Padova, Legnaro, Italy; Department of Agronomy, Food, Natural Resources, Animals and Environment—DAFNAE, University of Padova, Legnaro, Italy; Faculty of Agricultural, Environmental and Food Sciences, Free University of Bozen-Bolzano, Bolzano-Bozen, Italy; Competence Centre for Plant Health, Free University of Bozen-Bolzano, Bolzano-Bozen, Italy; Hawkesbury Institute for the Environment, Western Sydney University, Penrith, Australia; Hawkesbury Institute for the Environment, Western Sydney University, Penrith, Australia; Department of Agronomy, Food, Natural Resources, Animals and Environment—DAFNAE, University of Padova, Legnaro, Italy

**Keywords:** microbiota, dysbiosis, *Candidatus* Erwinia dacicola, *Bactrocera oleae*, IPM

## Abstract

The olive fly *Bactrocera oleae* (Rossi) is one of the most destructive olive pests, threatening olive and olive oil production worldwide. Traditionally managed with synthetic insecticides, its control now requires alternative strategies due to regulatory restrictions. The disruption of the obligate association between the pest and its primary symbiont *Candidatus* Erwinia dacicola, Capuzzo et al. (2005) has been reported following the application of a Zn–Cu–citric acid bio-complex compound (Dentamet) to olives infested with newly laid eggs. In this study, the effects of Dentamet exposure on the composition and structure of the *B. oleae* bacterial community were evaluated by comparing flies from treated (Dentamet-treated [DT]) olives, with untreated (non-treated [NT]) olives under laboratory conditions, and olives collected from untreated orchards (wild-in-controlled-environment). Bacterial communities were characterized by 16S rRNA gene sequencing on an Illumina MiSeq platform, and quantitative Real-Time PCR (qPCR) was used to estimate the abundance of *Ca.* E. dacicola and 4 additional bacterial genera involved in detoxification and nutrient metabolism. Metabarcoding analyses revealed differences between DT and NT samples, including reduced bacterial diversity in DT larvae and a shift in the adult bacterial community structure supported by beta diversity analyses. qPCR analysis showed a lower abundance of *Ca.* E. dacicola in DT larvae (significant), pupae, and adults, alongside decreases in the 4 investigated bacterial genera. Overall, these findings suggest that Dentamet exposure is associated with changes not only in the primary symbiont but also in the broader bacterial community, with potential implications for key microbial interactions.

## Graphical Abstract

**Graphical Abstract:**
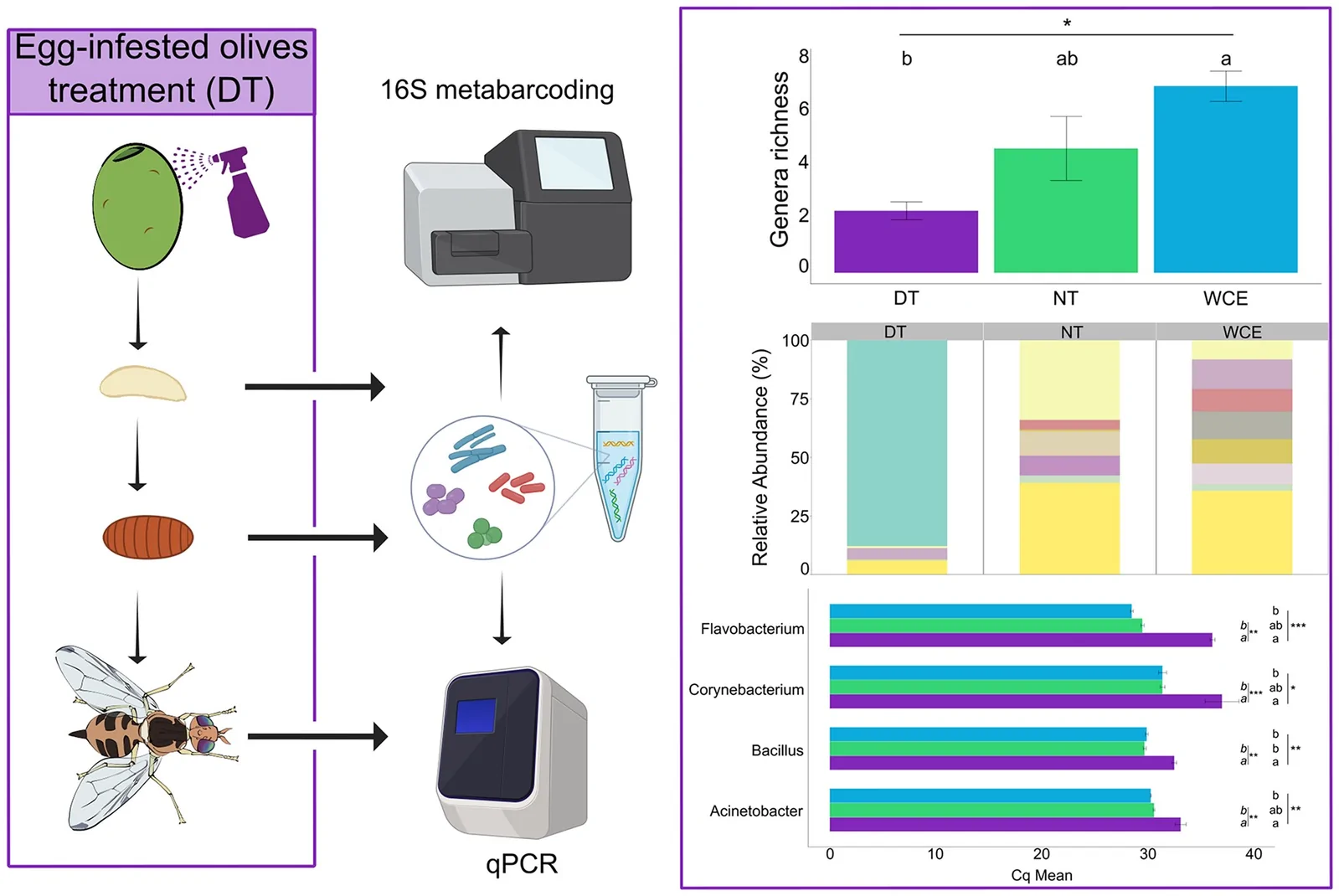

## Introduction

The olive fruit fly, *Bactrocera oleae* (Rossi) (Diptera, Tephritidae), is widely recognized as the most damaging pest affecting olive (*Olea europaea* L.) cultivation throughout the Mediterranean region, as well as in other olive-growing areas worldwide (Raspi and Viggiani 2008, Daane and Johnson 2010, Živković et al. 2025). This monophagous insect lays its eggs within the mesocarp of olive drupes, where larval development occurs through 3 instars. As the larvae feed internally, they compromise the integrity of the fruit, leading to premature drop, pulp degradation, and a subsequent oxidative deterioration of the resulting olive oil (Neuenschwander and Michelakis 1978, Malheiro et al. 2015, Taiti et al. 2025). The consequent economic losses are substantial and are often compounded by the degradation of key quality parameters in virgin olive oil, such as increased free acidity, elevated peroxide values, and reduced phenolic content (Gomez-Caravaca et al. 2008, Gucci et al. 2012, Stavrianakis et al. 2025).

Historically, the management of *B. oleae* infestations has relied heavily on broad-spectrum insecticides, among which the systemic organophosphate dimethoate was one of the most widely applied due to its efficacy and its relatively low persistence in olive oil (Cabras et al. 1997, Skouras et al. 2007, Kumral et al. 2020). However, growing awareness of its environmental and toxicological impact, coupled with regulatory changes, resulted in the ban of dimethoate in the European Union (EU Regulation 2020/1643) (Ortis et al. 2025). The lack of efficient insecticides resulted in an urgent need to explore alternative control options grounded in ecological sustainability and host-specific vulnerabilities.

The olive fruit fly harbors *Candidatus* Erwinia dacicola (hereafter *Ca.* E. dacicola), an obligate primary endosymbiotic bacterium that is vertically transmitted and located both in the midgut and within a specialized cephalic organ known as the esophageal bulb (EB). During oviposition, females release symbiont cells via contractile perianal glands and smear them onto the egg surface, thereby ensuring transgenerational transmission (Girolami 1973, Capuzzo et al. 2005, Bigiotti et al. 2021). This coordinated mechanism enables the establishment of the symbiosis early in larval development and highlights the essential role of *Ca.* E. dacicola in supporting *B. oleae* development, as it contributes to nitrogen assimilation and to the detoxification of phenolic compounds within the olive drupes, an environment inherently limited in available nitrogen and rich in phenolics (Ben-Yosef et al. 2015, Siden-Kiamos et al. 2022).

In addition to this primary symbiont, the olive fruit fly harbors gut microbial communities comprising taxa with metabolic, immunological, and ecological functions that contribute to host fitness and adaptation (Ben-Yosef et al. 2015, Blow et al. 2020). The integration of these symbiotic relationships into key physiological functions has positioned the olive fly’s microbiota as a central target for innovative pest control approaches. It is anticipated that insects that rely on environmentally acquired or egg-smeared symbionts are particularly vulnerable to interventions targeting early symbiont acquisition, thus providing a potential target for symbiotic control approaches (Gonella et al. 2020).

A recent study showed that impairing symbiont transmission in *B. oleae* by dipping egg-infested olives into antimicrobial agents led to significant reductions in symbiont load and corresponding decreases in larval development and pupation rates (Perin et al. 2023). Among the tested agents, a (4%)Zn–(2%)Cu–(23.8%) citric acid biocomplex, commercially available as Dentamet (Diachem, Italy), originally developed as a micronutrient EC foliar fertilizer in olive groves, has emerged as a particularly promising candidate. This formulation combines documented antimicrobial properties (Perin et al. 2023), which are likely attributable to the presence of copper ions, known for their broad-spectrum activity against bacterial membranes and metabolic processes, with demonstrated oviposition-deterrent effects against *B. oleae* (Checchia et al. 2022).

The present study was designed to investigate the impact of the Zn–Cu–citric acid biocomplex on the microbial community associated with *B. oleae* across all developmental stages. By integrating high-throughput 16S rRNA gene metabarcoding with quantitative Real-Time PCR (qPCR), we aimed to evaluate changes in bacterial community structure and symbiont abundance following exposure to this biocomplex. Particular attention was given to quantifying changes in the relative abundance of taxa within the core microbiota, as well as shifts in *Ca.* E. dacicola. To simulate realistic field conditions, the Zn–Cu–citric acid biocomplex was applied at concentrations and with an application method similar to standard open field treatments. Overall, our findings aim to deepen the current understanding of olive fruit fly-microbiota interactions and assess the prospects of microbiota-targeted interventions as part of integrated pest management frameworks for olive cultivation.

## Material and Methods

### Origin and Rearing of Olive Flies

Infested olives were manually collected from a pesticide-free olive grove located in Fossacesia (42°14′34.6″ N, 14°29′09.1″ E, Chieti, Abruzzo Region, Italy) in September 2023. The olives were transported to the laboratory of Entomology of the DAFNAE Department at the University od Padua to rear out adult flies from infested fruit. Newly emerged flies were transferred into cubical mesh cages (BugDorm, MegaView Science Co., Ltd., Taichung, Taiwan; side: 30 cm) to establish age-synchronized cohorts. The flies were fed a dry diet composed of sucrose and dried yeast extract (Sigma-Aldrich) in a 4:1 ratio, with water supplied via a sponge refreshed every 7 to 10 d.

### Experimental Design

Three experimental groups were established, each representing a different condition affecting the gut microbiota (Fig. 1). The first group, referred to as the wild-in-controlled-environment (WCE), was derived from mature larvae obtained from olives collected in the Fossacesia field and subsequently maintained under microbiologically controlled laboratory conditions, following the procedures described by Capuzzo et al. (2005) and Mazzon et al. (2010). Briefly, mature larvae emerging from olives were surface-sterilized with sodium hypochlorite (NaClO 1%), rinsed with sterile water, and allowed to pupate in sterilized sand. Pupae were re-sterilized, rinsed, and individually placed in sterile vials until adult emergence. All emerging adults were then collected and preserved in 96% ethanol at −20 °C about 24 h after emergence, until further analysis. Obtaining adults from surface-sterilized pupae and keeping them in axenic conditions allowed us to minimize the risk of microbiota contamination by free-living environmental bacteria.

**Fig. 1.**
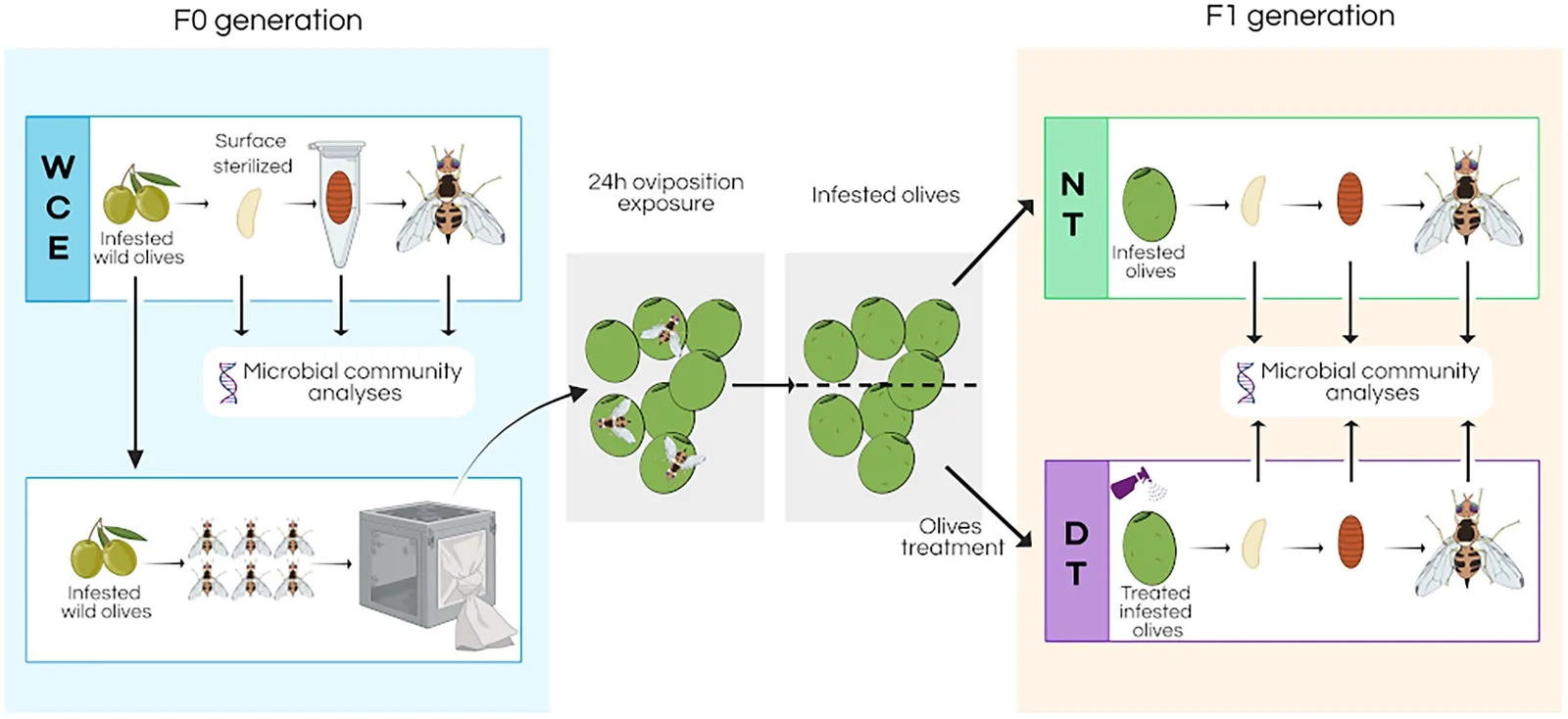
Experimental design illustrating the establishment of the 3 evaluated experimental groups. The wild-in-controlled-environment (WCE) group (F_0_ generation) was obtained from mature larvae emerging from field-collected infested olives, which were surface-sterilized and reared under microbiologically controlled laboratory conditions. Adults obtained from the same wild olives were used for oviposition to generate the F_0_ generation. After a 24-h oviposition period, infested olives were divided into 2 groups: non-treated (NT) and treated (DT). The DT fruits were treated with a Zn–Cu–citric acid biocomplex solution (5.47 ml/l, label-recommended dose) and dried under sterile conditions. Individuals from each experimental group were sampled at 3 developmental stages (larva, pupa, and adult) for downstream microbial community analyses.

The second group, designated as Dentamet-treated (DT), consisted of individuals reared from olives treated with Dentamet ([4%]Zn–[2%]Cu–[23.8%] citric acid biocomplex, hereafter Zn–Cu–citric acid biocomplex) 24 h after oviposition by lab-reared field individuals, while the third group, designated as non-treated (NT), comprised individuals that developed from untreated olives after oviposition by lab-reared field individuals (Fig. 1). For this, adult flies emerging from infested Fossacesia olives were allowed to oviposit in uninfested olives manually collected from an insecticide-free orchard in Cazzano di Tramigna (Verona, Veneto Region, Italy; 45°28′09.5″N, 11°12′37.5″E). Upon arrival at the laboratory, the fruits were carefully inspected to exclude any drupes showing signs of damage, decay, or pre-existing *B. oleae* infestation; moreover, a subset of olives was incubated as negative control to verify the absence of infestation. Then the selected olives were used as oviposition substrates for mature 10-d-old females. Exposure to oviposition lasted 24 h, after which 380 infested olives, showing a maximum of 3 oviposition punctures, confirmed under a stereomicroscope (Leica, Mannheim, Germany), were selected for the experiment. For the DT group, the 190 selected fruits were sprayed uniformly over the entire surface with the Zn–Cu–citric acid biocomplex at 5.47 ml/l concentration, which represents the label-recommended dose, until close to the point of runoff, then dried on mesh trays under a laminar flow hood at room temperature. Treated and untreated fruits were kept in separate containers according to the treatment and incubated at 20 to 25 °C to allow egg hatching and larval development.

Individuals from each group were sampled at 3 key life stages: third-instar larvae (10 d post-oviposition), pupae (13 d), and newly emerged adults (within 24 h after emergence). Sampled larvae and pupae were first rinsed with sterile distilled water, then stored in absolute ethanol, and preserved at −20 °C until further processing. From each adult, the esophageal bulb (EB) and midgut (G), representing distinct compartments of the digestive tract, were carefully dissected. All procedures were performed under a stereomicroscope in a laminar flow hood using sterile instruments. Prior to dissection, flies were immobilized by keeping flies in vials at 4 °C for 20 min, then surface-sterilized by rinsing in a 1% sodium hypochlorite solution for 2 min, followed by at least 2 rinses in sterile water. For EB extraction, the head was separated from the body, and the EB was isolated by splitting the head capsule into 2 parts starting from the occipital foramen. For G extraction, the abdomen was opened, the whole intestine was extracted, and the G tract was obtained by sectioning between the cardiac valve and the Malpighian tubules. Each EB and G sample was preserved in absolute ethanol in a sterile microcentrifuge tube and stored at −80 °C.

### Genomic DNA Extraction and 16S rRNA Gene Amplicon Sequencing

Total genomic DNA was extracted from 36 samples, each consisting of an independent pool of 3 specimens. For each life stage (larva, pupa, adult-EB, adult-G) and experimental condition (WCE, DT, NT), 3 pooled samples were processed using the DNeasy Blood & Tissue Kit (Qiagen, Hilden, DE), following the manufacturer’s instructions. DNA yield was evaluated using a Qubit 4.0 fluorometer (Thermo Fisher Scientific, Carlsbad, California, United States), paired with a Qubit 1× dsDNA High Sensitivity Quantification Assay Kit (Thermo Fisher Scientific, Carlsbad, California, United States).

Library preparation and sequencing of the v3 to v4 hypervariable region of the 16S rRNA gene were carried out by Macrogen Inc. (Seoul, South Korea). Briefly, the 16S rRNA gene metabarcoding libraries were prepared using the Nextera XT DNA Library Preparation Kit (Illumina Inc., San Diego, California, United States), following the Illumina 16S rRNA gene Metagenomic Sequencing Library protocol. The v3 to v4 hypervariable region was targeted using the 341F and 805R universal primers (Herlemann et al. 2011). Sequencing was conducted on a MiSeq platform (Illumina Inc., San Diego, California, United States), generating paired-end reads suitable for downstream microbial community analyses.

Raw sequencing data were processed in R (v4.4.1, “Race for Your Life”) (R Core Team 2024) using the “dada2” package (v1.34.0) (Callahan et al. 2016). Initial quality inspection of both forward and reverse reads was carried out using the *plotQualityProfile()* function to visually assess base quality across read positions. Based on these profiles, forward and reverse reads were quality-filtered and truncated at 260 and 220 bp, respectively, using the *filterAndTrim()* function to remove low-quality tails and discard reads with ambiguous bases. The resulting high-quality reads were then dereplicated with *derepFastq()*, collapsing identical sequences into unique representatives while retaining abundance information. Denoising was performed with the *dada()* function, which applies a probabilistic error model to distinguish biological variations from sequencing, resulting in Amplicon Sequence Variants (ASVs). Denoised forward and reverse reads were subsequently merged using *mergePairs()*. To eliminate potential artifacts introduced during PCR amplifications, chimeric sequences were identified and removed using the *removeBimeraDenovo()* function. Taxonomic assignment was performed using the *assignTaxonomy()* function, which implements a naïve Bayesian classifier to compare ASVs against the SILVA SSU reference database (v138.2) (Quast et al. 2013, Callahan 2024). Following taxonomic annotation, ASVs assigned to chloroplast or mitochondrial origin were removed from the dataset to exclude non-bacterial sequences from downstream analyses.

To minimize bias introduced by uneven library sizes, Cumulative Sum Scaling normalization was performed prior to the calculation of alpha- and beta-diversity metrics. Subsequent processing of the ASV count matrix and corresponding taxonomic assignments was conducted in RStudio (v2025.05.0 + 496 “Mariposa Orchid”), using a combination of the *tibble* (Müller and Wickham 2023) and *TaxaPhyloseq* (McMurdie and Holmes 2013) packages for data structuring and manipulation. Microbial diversity analyses were performed with the *MicrobiotaProcess* package (Xu et al. 2023). Alpha diversity was assessed by calculating genus-level richness, Shannon diversity index, and Simpson’s evenness index. Beta diversity was assessed by calculating the Bray–Curtis dissimilarity matrix visualized using Principal Coordinates Analysis (PCoA), and, for compositional comparison, by calculating the Euclidean distance matrix derived from Centered Log-Ratio (CLR)-transformed data, visualized using Principal Component Analysis (PCA). Prior to CLR transformation, zero values in the ASVs table were replaced using the Count Zero Multiplicative method using the *zCompositions* package (Palarea-Albaladejo and Martín-Fernández 2015). PCoA and PCA served as complementary ordination techniques to evaluate the consistency of group separation patterns across distance metrics. Differences in community composition among groups were statistically tested using PERMANOVA and ANOSIM, while group dispersion was evaluated with PERMDISP. When the global test indicated a significant overall effect, pairwise comparisons within groups were conducted, with *P*-values adjusted for multiple comparisons using the Bonferroni correction, applied separately within each test and distance metric. All plots were generated using the *ggplot2* package (Wickham 2016).

To further investigate potential differences among microbiota across the 3 experimental conditions (WCE, DT, and NT), alpha and beta diversity analyses were also performed after excluding the sequences of the primary symbiont *Ca.* E. dacicola. This approach allowed a more detailed assessment of shifts within the lower-abundance bacterial community.

The number of independent biological replicates was limited by sample availability; therefore, the analyses were conducted with a relatively small sample size, potentially affecting statistical power. This aspect was taken into account when interpreting the results.

### Quantification of Core Bacterial Genera

To quantify bacterial abundance, quantitative Real-Time PCR (qPCR) assays were performed on the same genomic DNA using a QuantStudio 6 platform (Thermo Fisher Scientific, Carlsbad, California, United States). These assays targeted the primary symbiont *Ca.* E. dacicola and 4 bacterial genera, *Acinetobacter*, *Bacillus*, *Corynebacterium*, and *Flavobacterium* that were found within the microbiota of *B. oleae* analyzed in this study (see Results below). Primer pairs for these 4 genera were designed in NCBI Primer-BLAST (Ye et al. 2012) using sequences derived from the metabarcoding dataset generated in this study (Supplementary Table S1) and were subsequently tested in silico to ensure specificity and to exclude any potential off-target amplification.

Every reaction mix was set up in a 10 µl final reaction volume, using 5 µl of PowerUp SYBR Green Master Mix (Thermo Fisher Scientific, Carlsbad, California, United States), 300 nM of each forward and reverse primer, and 2 µl of gDNA. Primer specificity for the newly designed assays was tested experimentally through melt curve analysis (60 to 95 °C, 0.5 °C increments) to confirm the presence of a single amplification product. Amplification efficiency for each of the 4 primer assays was determined using a 6-point (*Acinetobacter* and *Flavobacterium*) or a 7-point (*Bacillus* and *Corynebacterium*) 10-fold serial dilution series of gDNA. Amplification efficiency was calculated from the slope of the resulting standard curve as E = 10^(−1/slope) − 1. All 4 assays showed amplification efficiencies within the acceptable range of 90% to 110% (Supplementary Table S1). Relative quantification of the primary symbiont and the 4 targeted bacterial genera was performed using the 2^−ΔΔCq^ method (Livak and Schmittgen 2001) and the *β-actin* gene of *B. oleae* as the reference gene (Livadaras et al. 2021). Non-template controls were included in each qPCR run to exclude contamination.

All statistical analyses were performed in RStudio using the *dplyr* package (Wickham et al. 2023). Significant differences among group means were evaluated using either one-way ANOVA with Tukey’s HSD test or the nonparametric Kruskal–Wallis test with Dunn’s test with Bonferroni correction, depending on the data distribution.

## Results

### Composition and Diversity of the *B. oleae* Microbiota

The metabarcoding analysis of the 16S rRNA gene yielded a total of 5,675,032 paired-end reads. After quality filtering and denoising with the dada2 pipeline, 5,224,252 high-quality paired-end reads, with an average merged sequence length of 409 bp, were retained for downstream analyses. The 154 identified ASVs were classified into 87 reference-based bacterial taxa. Overall, 98.1% of the annotated sequences were classified at phylum level, 96.8% at class level, 96.1% at order level, 90.3% at family level, and 89.6% at genus level, at which all subsequent analyses were conducted to maximize the taxonomic resolution for this dataset. To explore whether these taxonomic differences translated into broader patterns of microbial community structure and diversity, we assessed alpha and beta diversity metrics across developmental stages and treatments. Patterns in alpha (Supplementary Table S2) and beta diversity analyses showed that the gut microbiota of *B. oleae* responded to the Zn–Cu–citric acid biocomplex exposure in a stage-dependent manner.

Alpha diversity metrics in third-instar larvae revealed significant differences in genera richness (*P *= 0.017; Fig. 2A) and Simpson’s evenness (1/D × S) (*P *= 0.021; Fig. 2B) across DT, NT, and WCE groups. Genera richness was lowest in the DT group (2.33 ± 0.333), followed by the NT group (4.67 ± 1.20), and highest in WCE specimens (7.00 ± 0.577). In contrast, the evenness index was highest in the DT group (0.463 ± 0.066), decreased in the NT group (0.258 ± 0.066), and was lowest in the WCE group (0.159 ± 0.024). The Shannon diversity index (H′) did not differ among the groups, but showed a similar trend to genera richness, with the DT group showing the lowest value and the WCE group showing the highest value. In contrast, at the third-instar larval stage, no significant differences were observed in the beta diversity (PERMDISP *P *= 0.459, PERMANOVA *P *= 0.196). Both PCA and PCoA showed a visual trend toward tighter clustering of WCE specimens relative to a broader spread of DT and NT specimens (Fig. 3A and B); however, this pattern was not statistically supported. At the pupal stage, no significant differences in alpha diversity were observed across treatments. Similar to alpha diversity, beta diversity analyses revealed no significant differences across the 3 experimental conditions (PERMDISP *P *= 0.148 and PERMANOVA *P *= 0.245). At the adult stage, no significant differences were observed across richness or diversity indices, although a significant difference in sample dispersion was detected (PERMDISP *P *= 0.038) (Fig. 4A and B), with ordination analyses showing a broader spread of the DT specimens relative to a tighter cluster formed by the NT group.

**Fig. 2.**
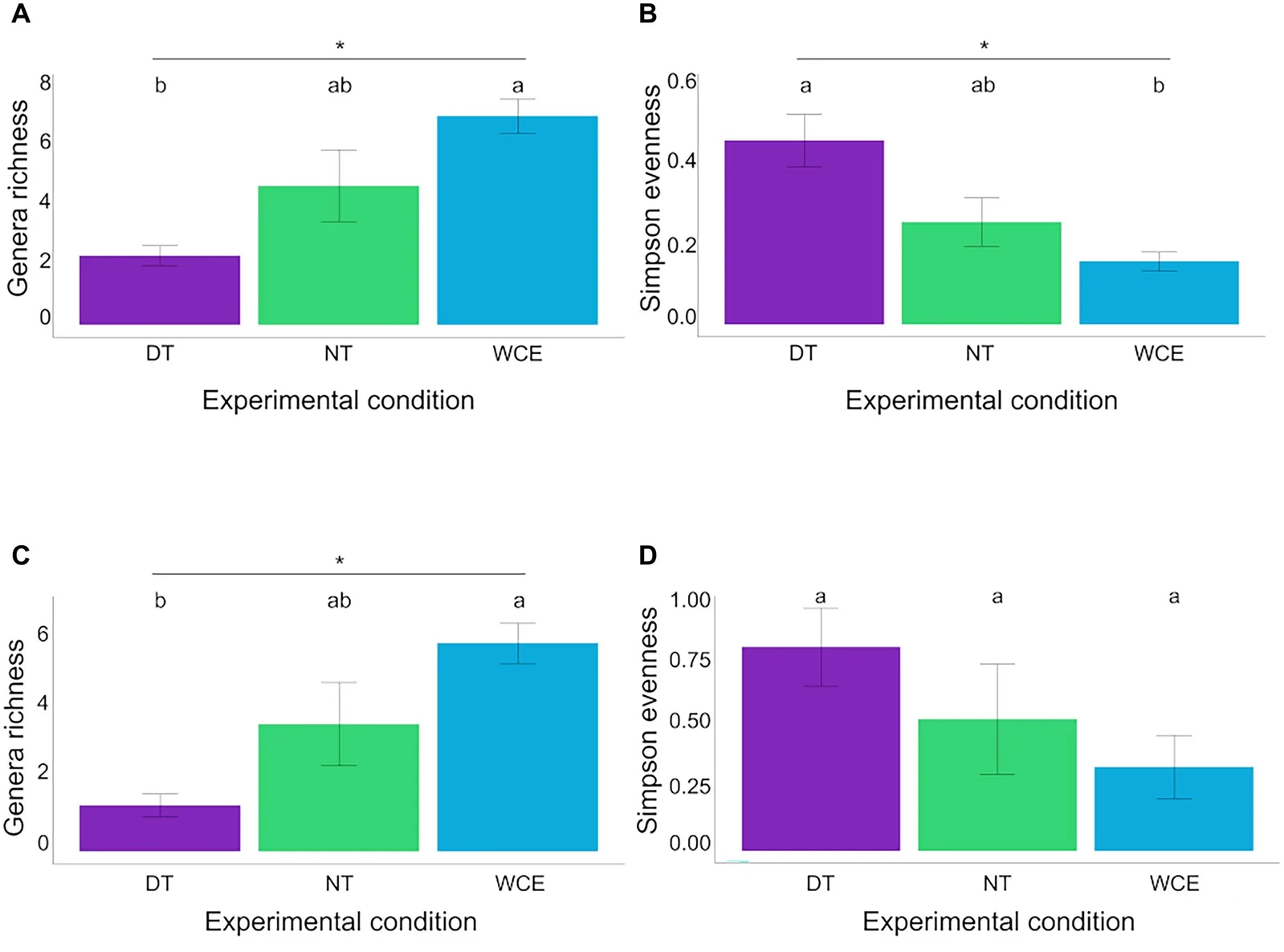
Barplot comparison to evaluate alpha diversity of DT specimens (purple), NT specimens (green), and WCE specimens (light blue) at the larval stage. A) Genera richness calculated including the primary symbiont *Ca.* E. dacicola in the dataset; B) Simpson’s evenness (1/D × S) index calculated including the primary symbiont *Ca.* E. dacicola in the dataset; C) genera richness calculated excluding the primary symbiont *Ca.* E. dacicola from the dataset; D) Simpson’s evenness (1/D × S) index calculated excluding the primary symbiont *Ca.* E. dacicola from the dataset. *Significance level *P* < 0.05.

**Fig. 3.**
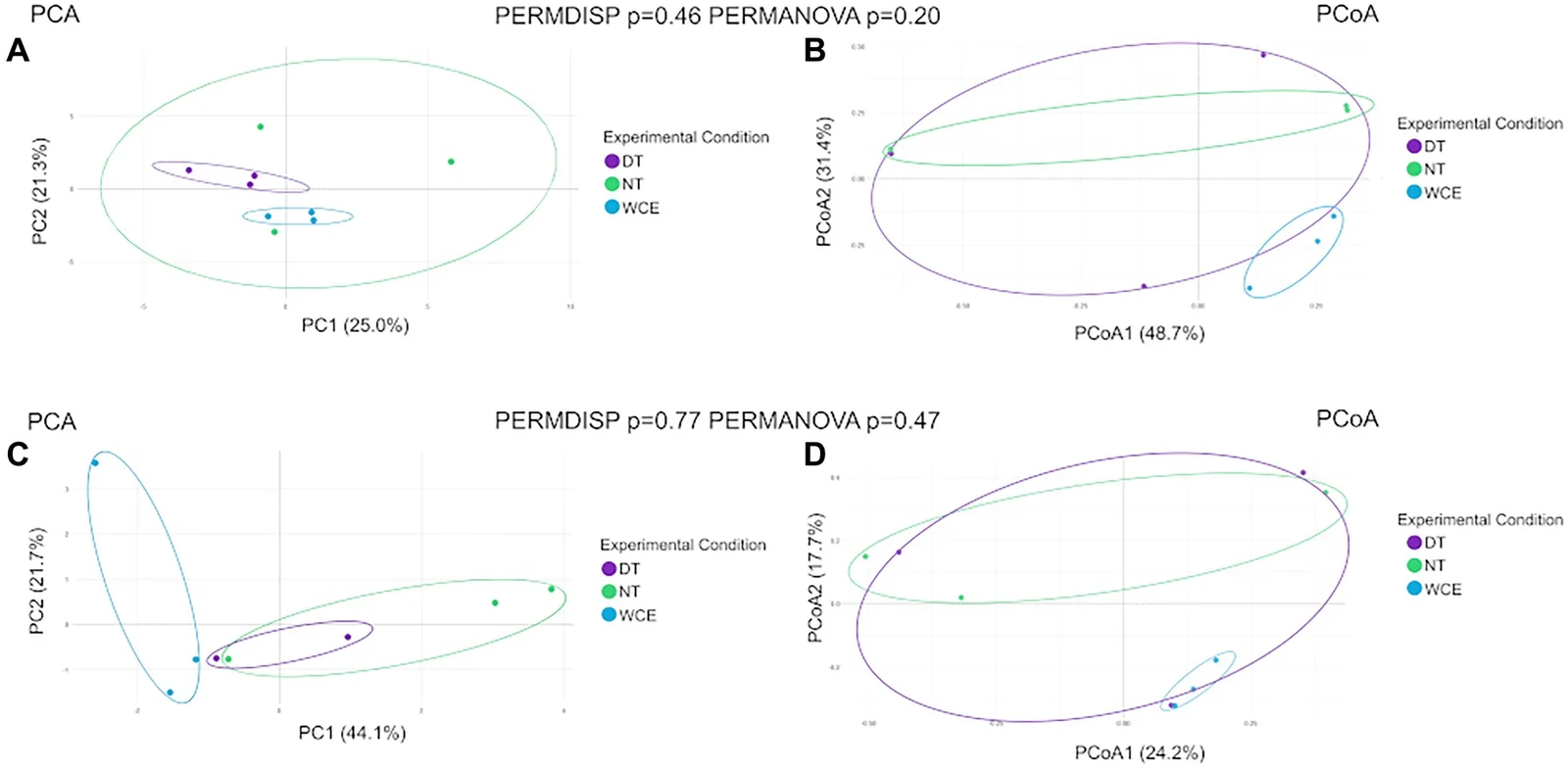
Multivariate analyses for the bacterial communities sequencing data at the genus taxonomic level to evaluate the beta diversity in DT specimens (purple), NT specimens (green), and WCE specimens (light blue) at the larval stage. A) Principal Component Analysis (PCA), the primary symbiont *Ca*. E. dacicola is included in the dataset; B) Principal Coordinates Analysis (PCoA) based on the Bray–Curtis dissimilarity matrix, the primary symbiont *Ca*. E. dacicola is included in the dataset, PERMDISP *P *= 0.46, PERMANOVA *P *= 0.20. C) Principal Component Analysis (PCA), the primary symbiont *Ca*. E. dacicola is excluded from the dataset; D) Principal Coordinates Analysis (PCoA) based on the Bray–Curtis dissimilarity matrix, the primary symbiont *Ca*. E. dacicola is excluded from the dataset, PERMDISP *P *= 0.77, PERMANOVA *P *= 0.47.

**Fig. 4.**
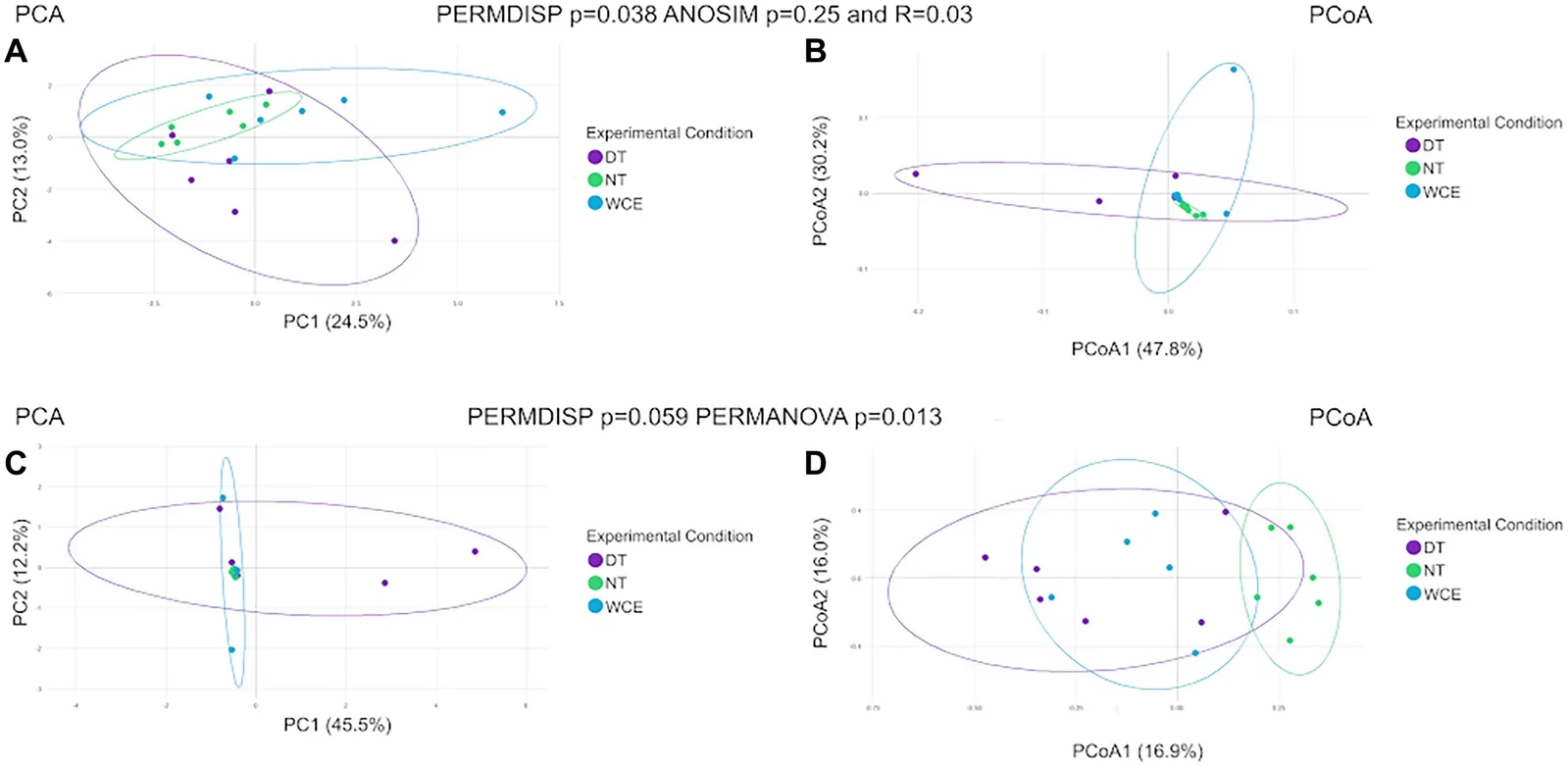
Multivariate analyses for the bacterial communities sequencing data at the genus taxonomic level to evaluate the beta diversity in DT specimens (purple), NT specimens (green), WCE specimens (light blue) at the adult stage. A) Principal Component Analysis (PCA), the primary symbiont *Ca.* E. dacicola is included in the dataset; B) Principal Coordinates Analysis (PCoA) based on the Bray–Curtis dissimilarity matrix, the primary symbiont *Ca*. E. dacicola is included in the dataset, PERMDISP *P *= 0.038, ANOSIM *P *= 0.25 and R = 0.03. C) Principal Component Analysis (PCA), the primary symbiont *Ca*. E. dacicola is excluded from the dataset; D) Principal Coordinates Analysis (PCoA) based on the Bray–Curtis dissimilarity matrix, the primary symbiont *Ca*. E. dacicola is excluded from the dataset, PERMDISP *P *= 0.059, PERMANOVA *P *= 0.013.

To further explore the differences among the microbiota associated with the experimental conditions, sequences assigned to *Ca.* E. dacicola, accounting for 97.8% of the total read counts in our dataset, were excluded from the analysis. At the larval stage, the significant differences in genera richness (*P *= 0.017) remained evident across the groups (Fig. 2C), with WCE specimens exhibiting the highest level of richness (6.00 ± 0.577), followed by NT (3.67 ± 1.20) and DT specimens (1.33 ± 0.333). Shannon diversity, Simpson’s evenness (Fig. 2D), and beta diversity metrics all indicated no significant differences in community structure (Fig. 3C and D, PERMDISP *P *= 0.770, PERMANOVA *P *= 0.473). At the pupal stage, both alpha (Supplementary Table S2) and beta diversity metrics (PERMDISP *P *= 0.675, PERMANOVA *P *= 0.183) remained non-significant across the experimental conditions. In adult specimens, alpha diversity indices did not reveal significant differences across treatments. Notably, beta diversity metrics uncovered significant differences in community composition (Fig. 4C and D, PERMANOVA *P *= 0.013), with ordination plots showing a more scattered DT group and a more tightly clustered NT group, reiterating patterns observed in the presence of the primary symbiont.

Although alpha and beta diversity metrics did not consistently yield significant differences within individual developmental stages and experimental conditions, the analysis of relative abundances of bacterial taxa revealed compositional shifts across treatments at all life stages (Supplementary Fig. S1). A closer examination of relative abundance patterns showed that, at the larval stage (Supplementary Fig. S1A), the microbiota of laboratory-reared specimens was largely represented by *Rosenbergiella*, which accounted for 97% of the relative abundance in DT larvae and 99.6% in NT larvae. In contrast, WCE larvae were primarily associated with *Enterobacter* (73.9%) and *Sphingobium* (17.3%). At the pupal stage (Supplementary Fig. S1B), DT specimens were characterized by *Pseudomonas* (29.8%), *Bradyrhizobium* (21.5%), and *Ligilactobacillus* (17.2%). NT pupae showed a marked predominance of *Rosenbergiella* (96.4%), whereas WCE pupae, by comparison, displayed a more balanced composition, with *Pantoea* (28.5%), *Rosenbergiella* (28.4%), and *Methylophilus* (17%) as the most abundant genera. In adults (Supplementary Fig. S1C), *Rosenbergiella* accounted for 88% of the relative abundance in the DT specimens, while NT adults' relative abundance was mainly composed of *Staphylococcus* (34%) and *Paucibacter* (10.4%). In WCE adults, the most abundant genera were *Pseudomonas* (12.7%), *Micrococcus* (11.9%), and *Streptococcus* (10.4%). Tissue-specific analyses further revealed distinct compositional profiles (Supplementary Fig. S1D). In the EB, DT specimens were characterized by *Rosenbergiella* (84%), NT specimens were primarily associated with *Prevotella* (23.3%) and *Staphylococcus* (18.4%), and WCE specimens showed higher relative abundances of *Micrococcus* (28.9%) and *Pseudomonas* (20.2%). In the midgut, DT specimens were predominantly composed of *Rosenbergiella* (89.4%), NT specimens were characterized by *Staphylococcus* (42.9%) and *Paucibacter* (16.4%), while WCE specimens were mainly associated with *Escherichia–Shigella* (16.4%), *Sutterella* (14.9%), and *Staphylococcus* (13.9%).

### Quantitative PCR-Based Quantification of Core Microbiota Members

Quantitative PCR analyses targeting 4 selected genera from the microbiota of *B. oleae*, i.e. *Acinetobacter*, *Bacillus*, *Corynebacterium*, and *Flavobacterium*, are reported in Fig. 5. These taxa were selected based on their putative functional roles, which overlap with those of the primary symbiont, particularly in nitrogen metabolism and the degradation of aromatic compounds (Prince and Cleverdon 1955, Sebastian et al. 1987, Tam et al. 2006, Peleg et al. 2012, Jung and Park 2015, Egamberdieva 2016, Saxena et al. 2020, Kim et al. 2023). Third-instar larvae exposed to the treatment exhibited a significant reduction in the log fold change of all 4 bacterial genera. At the pupal stage, the treatment resulted in a significant decrease in the log fold change of *Bacillus* and *Flavobacterium*, while no significant changes were observed for *Acinetobacter* and *Corynebacterium*. This pattern was maintained in adults, where the significant decrement in log fold change of *Bacillus* and *Flavobacterium* was observed in both the EB and midgut.

**Fig. 5.**
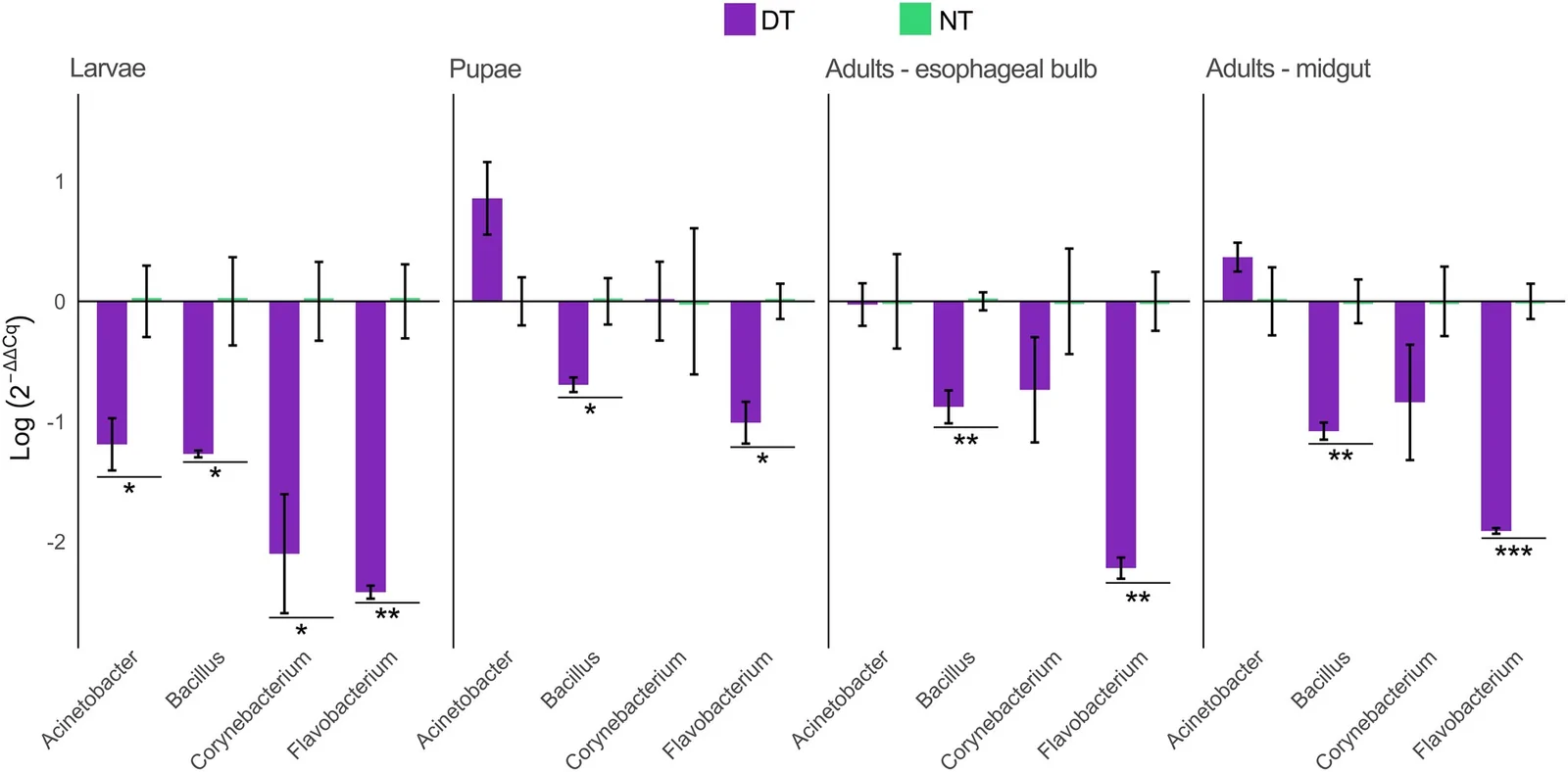
qPCR quantification of 4 core microbiota’s bacterial genera. The plot shows the comparison between the log fold change of DT specimens (purple) and NT Specimens (green) for all the tested genera at larval, pupal, and adult stage. *Significance level *P *< 0.05, **significance level *P *< 0.01, ***significance level *P *< 0.001.

qPCR results targeting the primary symbiont *Ca.* E. dacicola revealed an overall reduction in abundance across all developmental stages in the DT specimens (Fig. 6). At the larval stage, the DT group showed a significant reduction (*P *= 0.003) in the primary symbiont titer compared to the NT group (Fig. 6A). At the pupal stage, the DT specimens exhibited a trend, although non-significant, toward reduced primary symbiont abundance relative to NT specimens (Fig. 6B). In adult specimens, tissue-specific EB (Fig. 6C) and midgut (Fig. 6D) samples from treated specimens showed lower *Ca.* E. dacicola titers compared to NT controls, even though these differences were not statistically significant.

**Fig. 6.**
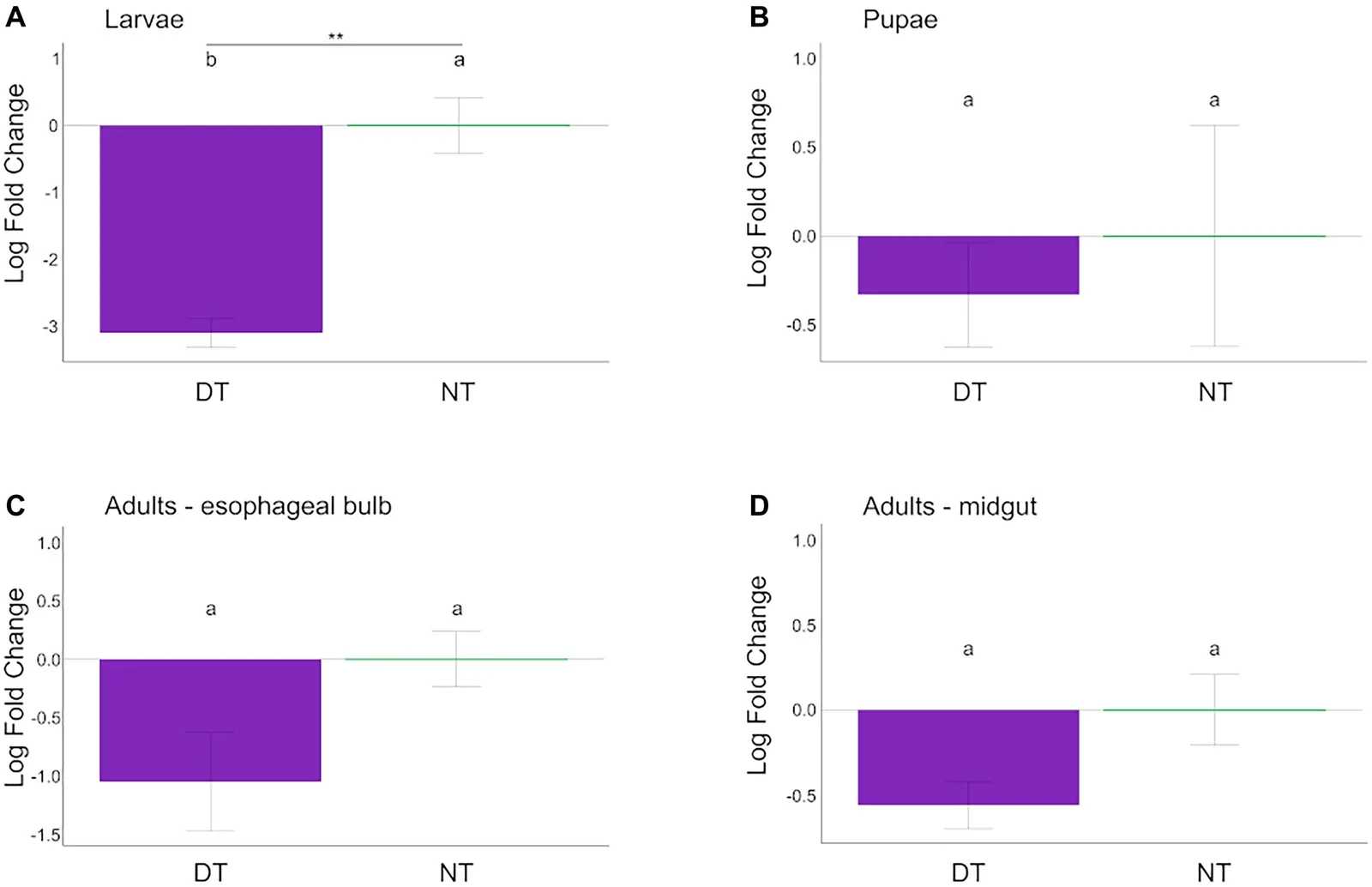
*Candidatus* E. dacicola quantification by qPCR. The plot shows the comparison between the log fold change of DT specimens (purple), and NT specimens (green). A) larval stage, B) pupal stage, C) adult stage–esophageal bulb, D) adult stage–midgut. Means with a different letter in the horizontal comparison are significantly different at the Kruskal–Wallis test. **Significance level *P *< 0.01.

## Discussion

The use of antimicrobial compounds to disrupt host–microbe associations has been previously investigated as a strategy to control several insect pests, such as the stink bugs *Halyomorpha halys* and *Nezara viridula* (Gonella et al. 2022, Gonella and Alma 2023, Prieto et al. 2023), as well as *B. oleae* (Perin et al. 2023). In this context, our study provides new insights into the microbiota-level effects of the Zn–Cu–citric acid biocomplex on *B. oleae* within the developmental stages of flies emerged from treated olives infested with eggs, as well as new evidence on bacterial taxa potentially capable of persisting through metamorphosis.

### Effects of Antimicrobial on Olive Fly Microbiota

The significant reduction in bacterial diversity observed in treated (DT) larvae, with genus richness almost 50% lower than in NT laboratory-reared group, is consistent with a treatment-associated shift in the bacterial community. This reduction in microbial richness aligns with previous reports showing that metal-based treatments can disrupt olive fly gut microbial communities, with consequent effects on host health and development (Bigiotti et al. 2019, Sinno et al. 2020, Malandrakis et al. 2024). Although the differences in Shannon diversity were not statistically significant, DT larvae showed a trend toward reduced overall bacterial complexity. Moreover, the higher evenness observed in the DT larvae indicates a shift towards a more homogenized community dominated by fewer taxa, possibly due to selective survival under Zn–Cu–citric acid biocomplex exposure. The absence of significant differences in alpha and beta diversity analyses at the pupal stage may indicate a stage-dependent attenuation of treatment effects, though it could also reflect the limited statistical power derived from the small sample size used in this study, which may constrain the ability to detect subtle community shifts. While stage-dependent microbiome resilience has been documented in other insects (Chouaia et al. 2019, Hammer and Moran 2019, Mason et al. 2023), disentangling genuine biological recovery from statistical detection limits remains beyond the resolution of the present dataset. Even though beta diversity did not consistently show significant treatment effects within individual developmental stages, the significant difference in dispersion observed in adults (PERMDISP *P *= 0.038) suggests that the early life-stage Zn–Cu–citric acid biocomplex exposure may influence the composition and stability of the gut microbial community. These changes seem to involve primarily the lower-abundant taxa, pointing to a stage-dependent pattern of compositional change.

### Effects of Antimicrobial on Olive Fly Microbiota Beyond the Primary Symbiont

Analyses of insect microbiota face a challenge arising from dominant bacterial symbionts that can overshadow the detection and analysis of less abundant microbial community members. This masking effect occurs when primary endosymbionts or highly prevalent facultative symbionts dominate sequencing datasets, thereby obscuring ecologically relevant but rare bacterial taxa (Schrieke et al. 2022). Such dominance has been widely documented across insect taxa. For example, in scuttle flies (Phoridae), bacterial loads varied by up to 4 orders of magnitude between individuals, with facultative symbionts such as *Wolbachia* and *Rickettsia* maintaining consistently high prevalence and abundance (Nowak et al. 2025). In Diptera, mosquito microbiomes are often dominated by *Wolbachia* or *Asaia*, resulting in rare bacterial taxa being frequently overlooked unless more refined analytical methods are applied (Schrieke et al. 2022). Our study provides a parallel case in *B. oleae*, where the primary symbiont *Ca.* E. dacicola accounted for 97.8% of the total sequences. Therefore, removing the overwhelmingly abundant primary symbiont enabled the detection of lower-abundance taxa that would otherwise remain obscured. This approach revealed that richness and compositional changes within the lower-abundance community were still detectable, suggesting that the antimicrobial treatment of infested olives is associated with shifts in the microbiota beyond those observed for the primary symbiont, potentially involving other significant core taxa as well.

Building on these refined analyses, the relative abundance data provided a more detailed view of the taxa contributing to the differences between laboratory-reared and WCE specimens, which align with established patterns in the literature (Blow et al. 2020). It should be highlighted that the bacterial taxa characterizing WCE specimens were most likely acquired prior to surface sterilization and axenic rearing, and therefore reflect a field-derived microbial legacy rather than an effect of the experimental condition itself. As such, WCE specimens cannot be considered as a wild control equivalent to NT, and the differences observed between WCE and laboratory-reared groups are attributable to the combined influence of field origin and axenic rearing history. The predominance of *Rosenbergiella* in laboratory-reared larvae (97% to 99.6%) contrasts with the diverse Enterobacteriaceae assemblage observed in WCE larvae, dominated by *Enterobacter* (73.9%). This is consistent with previous reports showing that wild *B. oleae* populations harbor diverse facultative Enterobacteriaceae, whereas laboratory conditions tend to favor simplified communities (Koskinioti et al. 2019, Blow et al. 2020, De Cock et al. 2020). The pupal stage further underscores developmental continuity within the microbiota, with taxa such as *Pseudomonas* and *Pantoea*, previously documented in adult *B. oleae* (Koskinioti et al. 2019), persisting across metamorphosis and reappearing in adult specimens, suggesting that their consistent presence may reflect functional roles within the bacterial community. Meanwhile, the detection of *Micrococcus* as a prominent genus in wild specimens (11.9% in adults) corroborates earlier reports including this genus in the lower-abundance component of the *B. oleae* microbiota (Campos et al. 2022). Moreover, the detection of *Sphingobium* (17.3% in WCE larvae) and *Methylophilus* (17% in WCE pupae) suggests that comprehensive sequencing can uncover previously overlooked taxa. Likewise, the presence of *Prevotella* (23.3% in NT EB) and *Sutterella* (14.9% in WCE midguts) expands knowledge of tissue-specific colonization patterns beyond the previously described *Acetobacter tropicalis* (Kounatidis et al. 2009) and *Tatumella* sp. TA1 (Blow et al. 2020), which have been reported as consistent symbionts of *B. oleae* but were not detected in this study. Moreover, the analysis of WCE adult specimens, derived from surface-sterilized larvae and emerged under sterile conditions, conducted without the dominance of *Ca.* E. dacicola, revealed additional bacterial taxa that would otherwise be obscured by the primary symbiont.

It is noteworthy that bacterial taxa such as *Micrococcus*, *Pseudomonas*, alongside *Acinetobacter*, *Corynebacterium*, *Bacillus*, and *Flavobacterium*, appear to be acquired during the larval stage and to persist into adulthood in the individuals reared under microbiologically controlled conditions (WCE specimens). Their persistence, as occurs for the primary symbiont, indicates their ability to endure the extensive structural reorganization taking place during pupal metamorphosis. This suggests a potential functional relevance of these taxa in metamorphic processes or in adult fitness (Hammer and Moran 2019, Campos et al. 2022). However, little is known about bacterial transmission throughout the different life stages of the olive fly, highlighting the need for further studies addressing symbiont continuity and turnover across development.

### Quantitative Evidence for Antimicrobial Treatment Effects

While community-level analyses provided valuable insights into structural shifts within the microbiota, a deeper understanding of how these changes may impact host-associated functions required targeted quantification of selected bacterial taxa. Accordingly, we complemented the metabarcoding results with qPCR assays targeting both the primary symbiont and other potentially relevant bacterial taxa. The decline in *Ca.* E. dacicola abundance observed at the larval stage highlights the sensitivity of this essential olive fly symbiont to environmental antimicrobial exposure. Conversely, the absence of significant differences in pupal and adult developmental stages suggests a partial recovery of symbiont load and may reflect a stage-dependent resilience of *Ca.* E. dacicola. As *Ca.* E. dacicola plays a central role in nitrogen assimilation and the detoxification of aromatic compounds such as oleuropein (Ben-Yosef et al. 2014, 2015), its depletion could potentially have adverse effects on fitness. Moreover, the analyses of the selected bacterial genera (i.e. *Acinetobacter*, *Bacillus*, *Corynebacterium*, and *Flavobacterium*) further revealed a significant treatment-associated reduction in relative abundance, particularly for *Bacillus* and *Flavobacterium*. These genera have documented associations with tephritid flies and, in other systems, with functions such as nitrogen metabolism and xenobiotic degradation; but the relationship between their reduction in *B. oleae* and tangible functional consequences remains hypothetical. *Acinetobacter* has been detected in wild *B. oleae* populations from Crete (Koskinioti et al. 2019) and has also been reported across other tephritid species in both larval and adult stages from wild and laboratory-reared populations (Malacrinò et al. 2018, Hadapad et al. 2019, De Cock et al. 2020, Bel Mokhtar et al. 2022). Although its specific functions within *B. oleae* have not been directly characterized, *Acinetobacter* is known for direct glucose oxidation (Duine 1991), aromatic compound degradation (Zhao et al. 2023), and nitrogen metabolism (Peleg et al. 2012). We therefore hypothesize that it could play analogous roles in nutrient transformation and detoxification in *B. oleae*. *Bacillus* has been isolated from a Spanish wild *B. oleae* population (Koskinioti et al. 2020) and detected in olive fruit flies from Greece and Italy (De Cock et al. 2020). In tephritids, *Bacillus* has been associated with diverse metabolic functions, including immunity (Raza et al. 2020), development (Akami et al. 2021), xenobiotic degradation (Xu et al. 2021), and nitrogen metabolism (Blencke et al. 2003), although whether it performs comparable functions in *B. oleae* has not been established. *Corynebacterium* has been consistently recorded as a persistent genus in wild *B. oleae* populations throughout the Mediterranean basin (Ben-Yosef et al. 2015, Campos et al. 2022). While its functions in *B. oleae* remain uncharacterized, a strain isolated from *Helicoverpa armigera* (Lepidoptera) was shown to degrade a plant defensive compound and improve host survival under chemical stress (Gu et al. 2022); this raises the hypothesis of a similar role in host adaptation in *B. oleae*, which remains to be tested directly. Finally, *Flavobacterium*, although previously reported in other *Bactrocera* species such as *Bactrocera jarvisi* and *Bactrocera tryoni* (Fitt and O’Brien 1985), is documented here for the first time in *B. oleae*. The functional role of *Flavobacterium* within the *Bactrocera* genus has not yet been investigated; however, members of this bacterial genus are known for their involvement in multiple nitrogen transformation processes (Behrendt et al. 2022, Choi et al. 2023), leading us to hypothesize a possible contribution to the host’s nitrogen balance in a nutrient-poor environment such as the olive drupe.

The simultaneous depletion of *Ca.* E. dacicola and auxiliary bacteria may thus compromise key physiological processes and reduce the microbiota’s functional resilience, with potential consequences for larval development and overall host fitness (Gould et al. 2018, Gupta and Nair 2020). However, further investigations regarding the functional relevance of these auxiliary taxa are warranted to better understand their contribution to the microbiota.

## Conclusions

While metabarcoding remains a valuable tool for profiling microbial communities, it is not always sufficient on its own to detect fine-scale or treatment-induced shifts in community composition (Lamb et al. 2019, Maretto et al. 2023). This limitation is particularly evident in host systems dominated by a primary symbiont, where the remaining microbiota tends to be low in richness and highly individualized. In such cases, increasing the number of biological replicates, could enhance statistical power and improve the ability to detect subtle yet biologically meaningful differences in diversity metrics or community structure. Given these constraints, the limited number of replicates used in this study, 3 pools of 3 individuals per investigated life stage, may have constrained the detection of subtle yet biologically relevant microbial shifts, as metabarcoding outcomes are highly sensitive to sample size (Nascimento et al. 2018). Nonetheless, our experimental design, which integrated complementary qualitative and quantitative approaches, provides an exploratory assessment of the microbial patterns and offers a basis for further investigations of the primary symbiont and, more broadly, of the associated microbial community.

It is important to reiterate that WCE specimens do not constitute a true wild control comparable to NT and DT: they differ both in origin (field-collected larvae versus laboratory-reared adults) and in rearing history (axenic rearing following surface sterilization versus conventional rearing), independently of antimicrobial exposure. Comparisons between WCE and NT/DT groups therefore reflect this combined effect of field origin and axenic rearing rather than treatment per se and should not be interpreted as evidence of treatment-driven divergence. With this caveat in mind, the overall comparison of WCE with laboratory-reared specimens nonetheless emphasizes the broader divergence between environmentally derived and laboratory-reared microbial communities, as previously observed by other authors (Voulgari-Kokota et al. 2022, Polidori et al. 2023). In this study, WCE larval specimens, although developed under sterile conditions, harbored a more taxonomically diverse microbiota, likely reflecting a bacterial legacy effect derived from their field origin. In contrast, NT specimens exhibited a distinct microbial profile shaped by laboratory conditions. These compositional differences highlight the role of environmental exposure in shaping the host microbiome and suggest that laboratory-based observations should be carefully contextualized, as they may slightly differ from microbiota dynamics in field populations.

Overall, our results highlighted the quantifiable effects of the Zn–Cu–citric acid biocomplex on the microbiota of *B. oleae*. The observed changes in both alpha and beta diversity metrics, along with the marked reduction in the primary symbiont, indicate a microbial impairment effect during the larval stage. The parallel reduction observed in core bacteria is consistent with this pattern, although the extent to which it contributes to a functional impairment remains hypothetical, as the roles of these taxa in *B. oleae* have not been functionally characterized. This early-life perturbation, as shown by qPCR results, appears to exert lasting effects that are not fully compensated by subsequent microbial restructuring in adult stages.

Further studies investigating microbial associates capable of persisting through metamorphosis, in addition to the primary symbiont *Ca.* E. dacicola, are warranted. Their persistence through metamorphosis suggests that they can constitute a selected fraction of the *B. oleae* microbiota that, to the best of our knowledge, remain largely unexplored despite their potential relevance for host development and fitness.

Taken together, these results provide an important foundation for understanding how antimicrobial inputs can reshape insect symbioses. Overall, our findings indicate that antimicrobial treatments are associated with compositional changes in the microbiota of *B. oleae* and with a marked depletion of the primary symbiont *Ca.* E. dacicola. The potential implications of these changes for host physiology remain to be determined. However, because 16S rRNA gene-based analyses do not allow direct inference of microbial functions, further studies integrating genomic and functional approaches are needed to clarify the biological consequences of the observed perturbations and the potential roles of the bacterial taxa involved. In particular, future work should assess whether these microbial changes translate into measurable effects on host fitness, thereby providing the basis for evaluating their potential relevance to integrated pest management strategies in olive orchards.

## Supplementary Material

ieag108_Supplementary_Data

## Data Availability

The dataset generated for this study can be found in the Sequence Read Archive (SRA), ID PRJNA1336730. https://www.ncbi.nlm.nih.gov/sra/PRJNA1336730

## References

[ieag108-B1] Ahmed M , Kim DR. 2018. pcr: an R package for quality assessment, analysis and testing of qPCR data. PeerJ. 6:e4473. 10.7717/peerj.4473 PMC5858653 29576953

[ieag108-B2] Akami M , Tamgue O, Ren X, et al 2021. Effects of symbiotic population impairment on microbiome composition and longevity of *Bactrocera dorsalis*. R Soc. Open Sci. 8:211104. 10.1098/rsos.211104 PMC8580452 34804572

[ieag108-B3] Behrendt U , Spanner T, Augustin J, et al 2022. Consumption of N2O by *Flavobacterium azooxidireducens* sp. nov. isolated from decomposing leaf litter of *Phragmites australis* (Cav.). Microorganisms 10:2304. 10.3390/microorganisms10112304 PMC9697520 36422374

[ieag108-B4] Bel Mokhtar N , Catalá-Oltra M, Stathopoulou P, et al 2022. Dynamics of the gut bacteriome during a laboratory adaptation process of the Mediterranean fruit fly, *Ceratitis capitata*. Front. Microbiol. 13:919760. 10.3389/fmicb.2022.919760 PMC9283074 35847076

[ieag108-B5] Ben-Yosef M , Pasternak Z, Jurkevitch E, et al 2014. Symbiotic bacteria enable olive flies (*Bactrocera oleae*) to exploit intractable sources of nitrogen. J. Evol. Biol. 27:2695–2705. 10.1111/jeb.12527 25403559

[ieag108-B6] Ben-Yosef M , Pasternak Z, Jurkevitch E, et al 2015. Symbiotic bacteria enable olive fly larvae to overcome host defences. R Soc. Open Sci. 2:150170. 10.1098/rsos.150170 PMC4632588 26587275

[ieag108-B7] Bigiotti G , Pastorelli R, Belcari A, et al 2019. Symbiosis interruption in the olive fly: effect of copper and propolis on *Candidatus Erwinia dacicola*. J. Appl. Entomol. 143:357–364. 10.1111/jen.12614

[ieag108-B8] Bigiotti G , Sacchetti P, Pastorelli R, et al 2021. Bacterial symbiosis in *Bactrocera oleae*, an Achille’s heel for its pest control. Insect Sci. 28:874–884. 10.1111/1744-7917.12835 32519794

[ieag108-B9] Blencke HM , Homuth G, Ludwig H, et al 2003. Transcriptional profiling of gene expression in response to glucose in *Bacillus subtilis*: regulation of the central metabolic pathways. Metab. Eng. 5:133–149. 10.1016/S1096-7176(03)00009-0 12850135

[ieag108-B10] Blow F , Gioti A, Goodhead IB, et al 2020. Functional genomics of a symbiotic community: shared traits in the olive fruit fly gut microbiota. Genome Biol. Evol. 12:3778–3791. 10.1093/gbe/evz258 PMC6999849 31830246

[ieag108-B11] Cabras P , Angioni A, Garau VL, et al 1997. Persistence of insecticide residues in olives and olive oil. J. Agric. Food Chem. 45:2244–2247. 10.1021/jf960922e

[ieag108-B12] Callahan BJ. (2024) Silva taxonomic training data formatted for DADA2 (version 138.2). Zenodo. 10.5281/zenodo.14169026

[ieag108-B13] Callahan BJ , McMurdie PJ, Rosen MJ, et al 2016. DADA2: high-resolution sample inference from Illumina amplicon data. Nat. Methods. 13:581–583. 10.1038/nmeth.3869 PMC4927377 27214047

[ieag108-B14] Campos C , Gomes L, Rei FT, et al 2022. Olive fruit fly symbiont population: impact of metamorphosis. Front. Microbiol. 13:868458. 10.3389/fmicb.2022.868458 PMC9058165 35509306

[ieag108-B15] Capuzzo C , Firrao G, Mazzon L, et al 2005. 'Candidatus *Erwinia dacicola*’, a coevolved symbiotic bacterium of the olive fly *Bactrocera oleae* (Gmelin). Int. J. Syst. Evol. Microbiol. 55:1641–1647. 10.1099/ijs.0.63653-0 16014495

[ieag108-B16] Checchia I , Perin C, Mori N, et al 2022. Oviposition deterrent activity of fungicides and low-risk substances for the integrated management of *Bactrocera oleae* (Diptera, Tephritidae). Insects 13:363. 10.3390/insects13040363 PMC9028197 35447804

[ieag108-B17] Choi A , Cha IT, Lee KE, et al 2023. The role of *Flavobacterium enshiense* R6S-5-6 in the Wetland Ecosystem revealed by whole-genome analysis. Curr. Microbiol. 80:83. 10.1007/s00284-022-03157-0 PMC9867689 36680647

[ieag108-B18] Chouaia B , Goda N, Mazza G, et al 2019. Developmental stages and gut microenvironments influence gut microbiota dynamics in the invasive beetle *Popillia japonica* Newman (Coleoptera: Scarabaeidae). Environ. Microbiol. 21:4343–4359. 10.1111/1462-2920.14797 31502415

[ieag108-B19] Daane KM , Johnson MW. 2010. Olive fruit fly: managing an ancient pest in modern times. Annu. Rev. Entomol. 55:151–169. 10.1146/annurev.ento.54.110807.090553 19961328

[ieag108-B20] De Cock M , Virgilio M, Vandamme P, et al 2020. Comparative microbiomics of tephritid frugivorous pests (Diptera: Tephritidae) from the field: a tale of high variability across and within species. Front. Microbiol. 11:1890. 10.3389/fmicb.2020.01890 PMC7431611 32849469

[ieag108-B21] Duine JA. (1991) Energy generation and the glucose dehydrogenase pathway in Acinetobacter. In: Towner KJ, Bergogne-Bérézin E, Fewson CA, editors. The biology of Acinetobacter. Vol. 57. Springer. 10.1007/978-1-4899-3553-3_20

[ieag108-B22] Egamberdieva D. 2016. *Bacillus* spp.: a potential plant growth stimulator and biocontrol agent under hostile environmental conditions. In: Islam M, Rahman M, Pandey P, et al Bacilli and agrobiotechnology. Springer (DE). pp. 91–111. 10.1007/978-3-319-44409-3_5

[ieag108-B23] Fitt GP , O’Brien RW. 1985. Bacteria associated with four species of Dacus (Diptera: Tephritidae) and their role in the nutrition of the larvae. Oecologia 67:447–454. 10.1007/BF00384954 28311582

[ieag108-B24] Girolami V. 1973. Reperti morfo-istologici sulle batteriosimbiosi del Dacus oleae Gmelin e di altri ditteri tripetidi, in natura e negli allevamenti su substrati artificiali. Redia 54:269–294.

[ieag108-B25] Gomez-Caravaca AM , Cerretani L, Bendini A, et al 2008. Effects of fly attack (*Bactrocera oleae*) on the phenolic profile and selected chemical parameters of olive oil. J. Agric. Food Chem. 56:4577–4583. 10.1021/jf800118t 18522402

[ieag108-B26] Gonella E , Alma A. 2023. The role of symbiont-targeted strategies in the management of pentatomidae and tephritidae pests under an integrated vision. Agronomy 13:868. 10.3390/agronomy13030868

[ieag108-B27] Gonella E , Orrù B, Alma A. 2022. Symbiotic control of *Halyomorpha halys* using a microbial biopesticide. Entomologia 42:901–909. 10.1127/entomologia/2022/1707

[ieag108-B28] Gonella E , Orrù B, Marasco R, et al 2020. Disruption of host-symbiont associations for the symbiotic control and management of pentatomid agricultural pest—a review. Front. Microbiol. 11:547031. 10.3389/fmicb.2020.547031 PMC7728854 33329418

[ieag108-B29] Gould AL , Zhang V, Lamberti L, et al 2018. Microbiome interactions shape host fitness. Proc. Natl. Acad. Sci. U. S. A. 115:E11951–E11960. 10.1073/pnas.1809349115 PMC6304949 30510004

[ieag108-B30] Gu M , Xue Z, Lv S, et al 2022. Corynebacterium sp. 2-TD mediated toxicity of 2-tridecanone to *Helicoverpa armigera*. Toxins. (Basel) 14:698. 10.3390/toxins14100698 PMC9609335 36287967

[ieag108-B31] Gucci R , Caruso G, Canale A, et al 2012. Qualitative changes of olive oils obtained from fruits damaged by *Bactrocera oleae* (Rossi). Horts. 47:301–306. 10.21273/HORTSCI.47.2.301

[ieag108-B32] Gupta A , Nair S. 2020. Dynamics of insect–microbiome interaction influence host and microbial symbiont. Front. Microbiol. 11:1357. 10.3389/fmicb.2020.01357 PMC7333248 32676060

[ieag108-B33] Hadapad AB , Shettigar SKG, Hire RS. 2019. Bacterial communities in the gut of wild and mass-reared *Zeugodacus cucurbitae* and *Bactrocera dorsalis* revealed by metagenomic sequencing. BMC Microbiol. 19:282. 10.1186/s12866-019-1647-8 PMC6929459 31870295

[ieag108-B34] Hammer TJT , Moran N. 2019. Links between metamorphosis and symbiosis in holometabolous insects. Philos. Trans. R Soc. Lond. B Biol. Sci. 374:20190068. 10.1098/rstb.2019.0068 PMC6711286 31438811

[ieag108-B35] Herlemann DPR , Labrenz M, Jürgens K, et al 2011. Transitions in bacterial communities along the 2000 km salinity gradient of the Baltic Sea. ISME J. 5:1571–1579. 10.1038/ismej.2011.41 PMC3176514 21472016

[ieag108-B36] Jung J , Park W. 2015. *Acinetobacter* species as model microorganisms in environmental microbiology. Appl. Microbiol. Biotechnol. 99:2533–2548. 10.1007/s00253-015-6439-y 25693672

[ieag108-B37] Kim M , Cha I, Lee K, et al 2023. Pangenome analysis of the genus *Flavobacterium*. Microbiol. Spectr. 11:e01003-23. 10.1128/spectrum.01003-23 PMC10655711 37594286

[ieag108-B38] Koskinioti P , Ras E, Augustinos AA, et al 2019. The effects of geographic origin and antibiotic treatment on the gut symbiotic communities of *Bactrocera oleae* populations. Entomologia Exp. Applicata. 167:197–208. 10.1111/eea.12764

[ieag108-B39] Koskinioti P , Ras E, Augustinos AA, et al 2020. Manipulation of insect gut microbiota towards the improvement of *Bactrocera oleae* artificial rearing. Entomologia Exp. Applicata. 168:523–540. 10.1111/eea.12934

[ieag108-B40] Kounatidis I , Crotti E, Sapountzis P, et al 2009. *Acetobacter tropicalis* is a major symbiont of the olive fruit fly (*Bactrocera oleae*). Appl. Environ. Microbiol. 75:3281–3288. 10.1128/AEM.02933-08 PMC2681620 19304818

[ieag108-B41] Kumral YA , Kumral NA, Kolcu A, et al 2020. Simulation study for the degradation of some insecticides during different black table olive processes. ACS Omega 5:14164–14172. 10.1021/acsomega.0c01907 PMC7301588 32566884

[ieag108-B42] Lamb PD , Hunter E, Pinnegar JK, et al 2019. How quantitative is metabarcoding: a meta-analytical approach. Mol. Ecol. 28:420–430. 10.1111/mec.14920 PMC7379500 30408260

[ieag108-B43] Livadaras I , Koidou V, Pitsili E, et al 2021. Stable inheritance of *Candidatus Erwinia dacicola* in olive fruit fly. Bull. Entomol. Res. 111:379–384. 10.1017/S0007485321000031 33541447

[ieag108-B44] Livak KJ , Schmittgen TD. 2001. Analysis of relative gene expression data using real-time quantitative PCR. Methods 25:402–408. 10.1006/meth.2001.1262 11846609

[ieag108-B45] Malacrinò A , Campolo O, Medina RF, et al 2018. Instar- and host-associated differentiation of bacterial communities in the Mediterranean fruit fly *Ceratitis capitata*. PLoS One. 13:e0194131. 10.1371/journal.pone.0194131 PMC5843337 29518170

[ieag108-B46] Malandrakis AA , Varikou K, Kavroulakis Ν, et al 2024. Copper nanoparticles interfere with insecticide sensitivity, fecundity and endosymbiont abundance in olive fruit fly *Bactrocera oleae* (Diptera: Tephritidae). Pest Manag. Sci. 80:3640–3649. 10.1002/ps.8068 38456555

[ieag108-B47] Malheiro R , Casal S, Baptista P, et al 2015. A review of *Bactrocera oleae* (Rossi) impact in olive products: from the tree to the table. Trends Food Sci. Technol. 44:226–242. 10.1016/j.tifs.2015.04.009

[ieag108-B48] Maretto L , Deb S, Ravi S, et al 2023. 16S metabarcoding, total soil DNA content, and functional bacterial genes quantification to characterize soils under long-term organic and conventional farming systems. Chem. Biol. Technol. Agric. 10:78. 10.1186/s40538-023-00450-3

[ieag108-B49] Mason CJ , Auth J, Geib SM. 2023. Gut bacterial population and community dynamics following adult emergence in pest tephritid fruit flies. Sci. Rep. 13:13723. 10.1038/s41598-023-40562-2 PMC10444893 37607978

[ieag108-B50] Mazzon L , Martinez-Sañudo I, Simonato M, et al 2010. Phylogenetic relationships between flies of the Tephritinae subfamily (Diptera, Tephritidae) and their symbiotic bacteria. Mol. Phylogenet. Evol. 56:312–326. 10.1016/j.ympev.2010.02.016 20171292

[ieag108-B51] McMurdie PJ , Holmes S. 2013. phyloseq: an R package for reproducible interactive analysis and graphics of microbiome census data. PLoS One. 8:e61217. 10.1371/journal.pone.0061217 PMC3632530 23630581

[ieag108-B52] Müller K , Wickham H. 2023. tibble: Simple data frames. R package. https://tibble.tidyverse.org/

[ieag108-B53] Nascimento FJA , Lallias D, Bik HM, et al 2018. Sample size effects on the assessment of eukaryotic diversity and community structure in aquatic sediments using high-throughput sequencing. Sci. Rep. 8:11737. 10.1038/s41598-018-30179-1 PMC6078945 30082688

[ieag108-B54] Neuenschwander P , Michelakis S. 1978. The infestation of *Dacus oleae* (Gmel.) (Diptera: Tephritidae) at harvest time and its influence on yield and quality of olive oil in Crete. Z. Angew. Entomol 86:420–433. 10.1111/j.1439-0418.1978.tb01948.x

[ieag108-B55] Nowak KH , Hartop E, Prus-Frankowska M, et al 2025. What lurks in the dark? An innovative framework for studying diverse wild insect microbiota. Microbiome 13:186. 10.1186/s40168-025-02169-9 PMC12341219 40796904

[ieag108-B56] Ortis G , Santoiemma G, Marangoni F, et al 2025. Efficacy of attract-and-kill techniques in controlling *Bactrocera oleae* (Diptera: Tephritidae). Insects 16:1161. 10.3390/insects16111161 PMC12653673 41302907

[ieag108-B57] Palarea-Albaladejo J , Martín-Fernández JA. 2015. zCompositions—R package for multivariate imputation of left-censored data under a compositional approach. Chemom. Intell. Lab. Syst. 143:85–96. 10.1016/j.chemolab.2015.02.019

[ieag108-B58] Peleg AY , de Breij A, Adams MD, et al 2012. The success of *Acinetobacter* species: genetic, metabolic and virulence attributes. PLoS One. 7:e46984. 10.1371/journal.pone.0046984 PMC3483291 23144699

[ieag108-B59] Perin C , Martinez-Sañudo I, Carofano I, et al 2023. Impairing the development of an olive fly pest by targeting its symbiotic bacteria in egg-infested fruits. Entomologia. (Pavia) 43:831–838. 10.1127/entomologia/2023/1916

[ieag108-B60] Polidori C , Ferrari A, Borruso L, et al 2023. Aedes albopictus microbiota: differences between wild and mass-reared immatures do not suggest negative impacts from a diet based on black soldier fly larvae and fish food. PLoS One. 18:e0292043. 10.1371/journal.pone.0292043 PMC10521979 37751428

[ieag108-B61] Prieto SV , Brunetti M, Magoga G, et al 2023. The intrinsic diversity of *Nezara viridula* gut symbionts affects the host fitness decline promoted by primary symbiont elimination. Entomologia 43:1151–1160. 10.1127/entomologia/2023/2224

[ieag108-B62] Prince HN , Cleverdon RC. 1955. The flavobacteria: II. Utilization of nitrogen compounds. J. Bacteriol. 69:307–309. 10.1128/jb.69.3.307-309.1955 PMC357532 14367278

[ieag108-B63] Quast C , Pruesse E, Yilmaz P, et al 2013. The SILVA ribosomal RNA gene database project: improved data processing and web-based tools. Nucleic Acids Res. 41:D590–D596. 10.1093/nar/gks1219 PMC3531112 23193283

[ieag108-B64] R Core Team. 2024. R: a language and environment for statistical computing. R Foundation for Statistical Computing.

[ieag108-B65] Raspi A , Viggiani G. 2008. On the senior authorship of *Musca oleae* (Diptera: Tephritidae). Zootaxa 1714:67–68. 10.11646/zootaxa.1714.1.7

[ieag108-B66] Raza MF , Yao Z, Bai S, et al 2020. Tephritidae fruit fly gut microbiome diversity, function and potential for applications. Bull. Entomol. Res. 110:423–437. 10.1017/S0007485319000853 32041675

[ieag108-B67] Saxena AK , Kumar M, Chakdar H, et al 2020. *Bacillus* species in soil as a natural resource for plant health and nutrition. J. Appl. Microbiol. 128:1583–1594. 10.1111/jam.14506 31705597

[ieag108-B68] Schrieke H , Maignien L, Constancias F, et al 2022. The mosquito microbiome includes habitat-specific but rare symbionts. Comput. Struct. Biotechnol. J. 20:410–420. 10.1016/j.csbj.2021.12.019 PMC8803474 35140881

[ieag108-B69] Sebastian J , Chandra AK, Kolattukudy PE. 1987. Discovery of a cutinase-producing *Pseudomonas* sp. cohabiting with a nitrogen-fixing *Corynebacterium* sp. J. Bacteriol. 169:131–136. 10.1128/jb.169.1.131-136.1987 PMC211744 3793714

[ieag108-B70] Siden-Kiamos I , Koidou V, Livadaras I, et al 2022. Dynamic interactions between the symbiont *Candidatus Erwinia dacicola* and its olive fruit fly host *Bactrocera oleae*. Insect Biochem. Mol. Biol. 146:103793. 10.1016/j.ibmb.2022.103793 35618174

[ieag108-B71] Sinno M , Bézier A, Vinale F, et al 2020. Symbiosis disruption in the olive fruit fly, *Bactrocera oleae* (Rossi), as a potential tool for sustainable control. Pest Manag. Sci. 76:3199–3207. 10.1002/ps.5875 32358914

[ieag108-B72] Skouras PJ , Margaritopoulos JT, Seraphides NA, et al 2007. Organophosphate resistance in olive fruit fly *Bactrocera oleae* populations in Greece and Cyprus. Pest Manag. Sci. 63:42–48. 10.1002/ps.1306 17103369

[ieag108-B73] Stavrianakis G , Sentas E, Zafeirelli S, et al 2025. Utilizing olive fly ecology towards sustainable pest management. Biology. (Basel) 14:125. 10.3390/biology14020125 PMC11851947 40001893

[ieag108-B74] Taiti C , Lombardo L, Godino G, et al 2025. Exploring morphological aspects, cuticle size and volatile compounds in the fruits of four olive cultivars as possibly interdependent components of *Bactrocera oleae* tolerance. Front. Plant Sci. 16:1681993. 10.3389/fpls.2025.1681993 PMC12698639 41393882

[ieag108-B75] Tam LT , Eyemann C, Albrecht D, et al 2006. Differential gene expression in response to phenol and catechol in *Bacillus subtilis*. Environ. Microbiol. 8:1408–1427. 10.1111/j.1462-2920.2006.01034.x 16872404

[ieag108-B76] Voulgari-Kokota A , Beukeboom LW, Wertheim B, et al 2022. Houseflies harbor less diverse microbiota under laboratory conditions but maintain a consistent set of host-associated bacteria. Sci. Rep. 12:11132. 10.1038/s41598-022-15186-7 PMC9249849 35778448

[ieag108-B77] Wickham H. 2016. ggplot2: elegant graphics for data analysis. Springer-Verlag.

[ieag108-B78] Wickham H , François R, Henry L, et al 2023. *dplyr: A Grammar of Data Manipulation*. https://dplyr.tidyverse.org, https://github.com/tidyverse/dplyr

[ieag108-B79] Xu S , Zhan L, Tang W, et al 2023. MicrobiotaProcess: a comprehensive R package for deep mining microbiome data. Innovation (Camb) 4:100388. 10.1016/j.xinn.2023.100388 PMC9988672 36895758

[ieag108-B80] Xu Y , Liu X, Zhao J, et al 2021. An efficient phthalate ester-degrading *Bacillus subtilis*: degradation kinetics, metabolic pathway, and catalytic mechanism of the key enzyme. Environ. Pollut. 273:116461. 10.1016/j.envpol.2021.116461 33485001

[ieag108-B81] Ye J , Coulouris G, Zaretskaya I, et al 2012. Primer-BLAST: a tool to design target-specific primers for PCR. BMC Bioinformatics 13:134. 10.1186/1471-2105-13-134 PMC3412702 22708584

[ieag108-B82] Zhao Y , Wei HM, Yuan JL, et al 2023. A comprehensive genomic analysis provides insights on the high environmental adaptability of Acinetobacter strains. Front. Microbiol. 14:1177951. 10.3389/fmicb.2023.1177951 PMC10149724 37138596

[ieag108-B83] Živković IP , Čirjak D, Hojsak L, et al 2025. Assessing cultivar-specific susceptibility and morphological adaptations of *Bactrocera oleae* (Diptera: Tephritidae) in olive orchards. J. Insect Sci. 25:1–11. 10.1093/jisesa/ieaf005 PMC11815492 39936610

