# Supplementary material for: Antimicrobial treatment of egg-infested olives is associated with perturbation and carry-over changes on bacterial communities in the olive fly Bactrocera oleae (Diptera, Tephritidae)
Source: J Insect Sci. 2026 Oct 3;26(5):ieag108. doi: 10.1093/jisesa/ieag108 (PMC13633668; doi:10.1093/jisesa/ieag108)
Supplement: Supplementary file 1 [file ieag108_supplementary_data.zip › Table S1.docx]

Table S1. Sequences of the primers used in this study

| Primer | Sequence | Reference |  |
| --- | --- | --- | --- |
| recA_F | TCTACCGGTTCGTTATCCCTG | Livadaras et al., 2021 |  |
| recA_R | AACGATAATCACGTCAACAGCAC |  |  |
| β-actin_F | CGGTATCCACGAAACCACAT | Livadaras et al., 2021 |  |
| β-actin_R | ATTGTTGATGGAGCCAAAGC |  |  |
| Primer | Sequence | NCBI Accession Number | PCR Efficiency |
| Corynebacterium_F | GACGAAGGGTTTCTGACGGT | MW020537.1 | E=97% R^2^=0.995 Slope=-3.41 |
| Corynebacterium_R | AGTTATGCCCGTATCGCCTG |  |  |
| Flavobacterium_F | TACCAATTGCGAAGGCAGGT | OQ834564.1 | E=102% R^2^=0.998 Slope=-3.28 |
| Flavobacterium_R | GCCACTGAGATTGCTCCCAA |  |  |
| Acinetobacter_F | GAAGGTCCCCTGCTTTCTCC | EU704859.1 | E=101% R^2^=0.999 Slope=-3.28 |
| Acinetobacter_R | TTGCTACTTGACCTAGCGGC |  |  |
| Bacillus_F | CCACACTGGGACTGAGACAC | KJ748584.1 | E=95% R^2^=0.995 Slope=-3.45 |
| Bacillus_R | CCGTGGCTTTCTGGTTAGGT |  |  |

The NCBI accession number is provided for all primers designed on the base of a reference sequence obtained from this work’s metabarcoding. PCR efficiency have been calculated using the *pcr* package for R (Ahmed and Kim, 2018).
