# Supplementary material for: Antimicrobial treatment of egg-infested olives is associated with perturbation and carry-over changes on bacterial communities in the olive fly Bactrocera oleae (Diptera, Tephritidae)
Source: J Insect Sci. 2026 Oct 3;26(5):ieag108. doi: 10.1093/jisesa/ieag108 (PMC13633668; doi:10.1093/jisesa/ieag108)
Supplement: Supplementary file 1 [file ieag108_supplementary_data.zip › Table S2.docx]

Table S2. Alpha-diversity metrics summary table

| Primary symbiont *Erwinia dacicola* included in the dataset | | | | | | | | | | |
| --- | --- | --- | --- | --- | --- | --- | --- | --- | --- | --- |
| Life Stage | Experimental Condition | Genera richness | | | Shannon diversity | | | Simpson evenness | | |
| Larva | DT | 2.33±0.333 | b | p=0.017 | 0.077±0.064 | a |  | 0.463±0.066 | a | p=0.021 |
|  | NT | 4.67±1.20 | ab |  | 0.110±0.067 | a |  | 0.258±0.066 | ab |  |
|  | WCE | 7.00±0.577 | a |  | 0.141±0.106 | a |  | 0.159±0.024 | b |  |
| Pupa | DT | 4.33±0.882 | a |  | 0.032±0.007 | a |  | 0.252±0.048 | a |  |
|  | NT | 6.00±2.00 | a |  | 0.228±0.203 | a |  | 0.281±0.113 | a |  |
|  | WCE | 6.00±1.53 | a |  | 0.103±0.046 | a |  | 0.194±0.041 | a |  |
| Adult | DT | 5.50±0.428 | a |  | 0.087±0.064 | a |  | 0.201±0.027 | a |  |
|  | NT | 6.83±1.14 | a |  | 0.009±0.002 | a |  | 0.165±0.023 | a |  |
|  | WCE | 4.83±1.30 | a |  | 0.035±0.025 | a |  | 0.366±0.139 | a |  |
| Primary symbiont *Erwinia dacicola* excluded from the dataset | | | | | | | | | | |
| Life Stage | Experimental Condition | Genera richness | | | Shannon diversity | | | Simpson evenness | | |
| Larva | DT | 1.33±0.333 | b | p=0.017 | 0.026±0.026 | a |  | 0.839±0.161 | a |  |
|  | NT | 3.67±1.20 | ab |  | 0.396±0.340 | a |  | 0.543±0.227 | a |  |
|  | WCE | 6.00±0.577 | a |  | 0.797±0.441 | a |  | 0.345±0.130 | a |  |
| Pupa | DT | 3.33±0.882 | a |  | 1.01±0.186 | a |  | 0.850±0.094 | a |  |
|  | NT | 5.00±2.00 | a |  | 0.423±0.379 | a |  | 0.487±0.261 | a |  |
|  | WCE | 5.00±1.53 | a |  | 0.807±0.192 | a |  | 0.377±0.057 | a |  |
| Adult | DT | 4.50±0.428 | a |  | 0.927±0.255 | a |  | 0.572±0.117 | a |  |
|  | NT | 5.83±1.14 | a |  | 1.54±0.205 | a |  | 0.788±0.036 | a |  |
|  | WCE | 4.60±1.29 | a |  | 1.11±0.298 | a |  | 0.769±0.099 | a |  |

Means with the same letter in the vertical comparison are not significantly different at the Kruskal-Wallis test
